# The relationship between co-occurring traumatic experiences and co-occurring mental health domains for veterans resident in Northern Ireland

**DOI:** 10.1186/s40359-024-01991-4

**Published:** 2024-10-01

**Authors:** Catherine Hitch, Erik Spikol, Paul Toner, Cherie Armour

**Affiliations:** 1 School of Psychology 1, Queen’s University Belfast, David Keir Building, 18-30 Malone Road, Belfast, BT9 5BN Northern Ireland, UK; 2 School of Psychology 2, Arden University, Middlemarch Park, Coventry, CV3 4FJ UK

**Keywords:** Veterans, Northern Ireland, Mental health, Trauma, Latent classes

## Abstract

**Introduction:**

Veterans residing in Northern Ireland (NI) likely experience higher levels of co-occurring lifetime trauma exposure and associated co-occurring mental health symptoms, due to the Troubles. As NI veterans have been subject to little exploration it is difficult to know how to support them. This exploratory study explored the co-occurrence of mental health symptoms as a function of co-occurring traumatic experiences.

**Methods:**

Two latent class analyses (LCA) were conducted on NI veteran data (*n* = 609). One LCA factored endorsements of 16-lifetime traumatic events, with a separate LCA exploring the co-occurrence of symptoms across six mental health domains. Mental health symptom classes were considered as a function of trauma classes, military-specific variables, gender and age.

**Results:**

Three trauma classes were identified: High Multi Trauma (10.84%); High Combat/Conflict (47.62%); Moderate Combat Conflict (41.54%), and three mental health symptom classes: High Co-occurring Mental Health (19.38%); High Depression Moderate Anxiety/Alcohol (24.63%); Moderate Alcohol/Normative (55.99%). Significant predictors of High Co-occurring Mental Health class membership were UDR service, ‘Worst’ military trauma and High Multi Trauma class membership. Both combat classes had a negative relationship with the High Co-occurring Mental Health. Predictors of the High Depression Moderate Anxiety/Alcohol class were High Multi Trauma class membership and UDR service, with Age and Moderate Combat Class membership having a negative relationship.

**Discussion:**

NI veterans could be labelled as ‘traumatised’ due to high levels of combat/conflict exposure, yet the two combat classes seemed unrelated to membership of poorer mental health classes. UDR membership indicated that living in one’s theatre of war could have mental health implications. It was concerning that 45% experienced some co-occurring mental health symptoms with 19% experiencing the poorest symptoms. Hazardous alcohol use appeared unrelated to poor mental health. Further research is needed, utilising robust methods. No clinical inferences are to be made from this exploratory study.

**Supplementary Information:**

The online version contains supplementary material available at 10.1186/s40359-024-01991-4.

## Introduction

Veteran literature notes the interplay between poor mental health symptoms and job-related traumatic event exposure, particularly for those who held combat roles [1]. Northern Ireland (NI) veterans could be a sub-population at risk of poorer mental health due to experiencing ‘systematic traumatic event layering’ across one’s lifetime [2]. Although NI veterans are under-researched, compared to other veteran populations, it is proposed they have experienced elevated levels of lifetime trauma exposure.

Many in NI began experiencing layered trauma exposure during childhood, including economic deprivation [3]. Such deprivation has led to some experiencing co-occurring adverse childhood experiences (ACEs), and resultant poorer mental health in adulthood [4].

Some veterans in NI grew up during an era of civil conflict. The history of sectarian violence in NI is complex but it could be described as long-running violent clashes between those aligned with the Protestant/Unionist identity/British Crown and those identifying as Catholic/Nationalist [5]. The violence escalated during the late 1960s, sparking the period referred to as the Troubles. Among other measures, physical ‘peace walls’ were erected in Belfast to reduce the effect of inter-community violence. Children growing up in NI at this time have been called ‘children of the Troubles’ to imply they were affected by ongoing sectarian community violence. Tomlinson [3] reported that children of the Troubles endorsed experiencing bombings, riots, a close relative’s death, assault (actual or witnessing), threat, and displacement. McLafferty and colleagues [4] found that children of the Troubles residing in NI were up to 15 times more likely to struggle with lifetime post-traumatic stress disorder (PTSD) and adulthood suicidiation.

If NI residents chose to join the military, regardless of where they served, they were often exposed to combat or military-specific traumatic events. During the Troubles, the ‘Homeserve’ Ulster Defence Regiment (UDR) was created as part of the peace-keeping mission called Operation BANNER (1969–2007). The UDR began as part-time, Reservist soldiers who were often deployed within their local neighborhoods [6]. The UDR lived and served within the same theatre of war, risking trauma exposure both in and out of uniform, as a representative of the Crown [6–8]. Non-native, Regular soldiers who were deployed to NI through Operation BANNER [9], were often confined to their barracks when off-duty for their safety.

The Troubles formally ended in 1998 but its legacy seems to continue, due to sectarian attitudes that persist in NI. During focus groups, veterans in NI described keeping their military affiliation secret as part of their safety hypervigilance. When asked about a preference for a dedicated NI veterans centre, veterans thought this would carry a security risk, because public perception towards them was still low [8, 10, 11]. Many veterans in NI appear to continue living with the effects of actual or perceived trauma exposure and choose to remain hidden.

Literature on the effects of trauma exposure on veteran mental health symptoms sometimes fails to account for the complexities of this relationship. For example, it cannot be assumed that all veterans experience PTSD [12]; many do not experience poor mental health due to exposure to trauma. Some veterans may have had a period of poor mental health but subsequently experienced post-traumatic growth [13]. Also, evidence suggests that if veterans experience poor mental health symptoms, their difficulties are likely to be comorbid. For example, Murphy and colleagues [14] found that 95.6% of their clinical UK veteran cohort had co-occurring mental health difficulties, with the predominant issue being PTSD. Yet, the predominance of PTSD may be shifting, as Williams et al., [15] reported more help-seeking UK veterans presented to the national charity Combat Stress with anxiety symptoms. Some who regularly support veterans in clinical settings have suggested complex PTSD (C-PTSD) is more prevalent than PTSD [16]. Additionally, many veterans who experience PTSD or C-PTSD may have accompanying symptoms of dissociation, caused by acute stress or traumatic exposure [17]. Armour and colleagues [18] found depression was the most frequently co-occurring difficulty endorsed by NI veterans within the general population. Finnegan and Randles [19] also noted depression was the most frequently coded mental health difficulty within UK veteran mental health records, followed by problematic alcohol. The high prevalence of alcohol use found by Murphy and colleagues [14] and Armour et al. [18] could be explained by the UK military’s historic relationship with alcohol use [20]. Some persistent predictors of poorer mental health, where military-related trauma has occurred, are gender (being female) and age (being younger) [21, 22].

A disproportionately high number of NI veterans, compared to the other three UK nations, have accessed mental health support through Combat Stress [23]. It was proposed that the number of referrals was due to military deployment within the Troubles and the paucity of mental health services available within NI [14]. If veterans engaged in help-seeking within NI they would find support services lacking, at both national and veteran population levels [7, 8]. The combination of lifetime trauma exposure, hypervigilance due to a perceived pending threat of trauma, and a lack of accessible/available support services could lead to the mental health landscape for NI veterans being particularly poor [24]. This may be most pertinent for members of the former UDR; they served and lived within the same theatre of war and could not escape actual or perceived traumatic event exposure. Threat perception research in NI suggests that threats of trauma exposure can be as detrimental to mental health as experiencing actual traumatic events [25].

There is no accurate understanding of the number of veterans living in NI, however, it is estimated there are approximately 40,000–60,000 [26, 27]. Historically, security concerns meant it was difficult to collect NI veteran-specific data [27], to assess the mental health landscape, and subsequently create or improve care. In an attempt to collect veteran-specific data, the Northern Ireland Veteran Health and Wellbeing Survey (NIVHWS) was launched in 2016 at a national (veteran) population level, collecting seminal data across a range of concepts. To date, the NIVHWS data has been used to investigate the symptom-level nuance of comorbid PTSD and alcohol difficulties via network analysis [28]. A further study conducted by Spikol and colleagues [29] utilised the NIVHWS data to explore latent class membership of PTSD and C-PTSD symptom endorsements. This study reported that a class containing PTSD was not found in the data. This finding was explained by the suggestion that populations exposed to higher levels of trauma are more likely to experience difficulties of self-organisation (DSO), being the key indicator for C-PTSD. Also, two out of three C-PTSD classes were predicted by anxiety, depression and summative traumatic events. The class specifics and their predictors demonstrated that the mental health landscape is nuanced in NI [29].

To date, the specific nature of co-occurring traumatic experiences and their association with a defined set of possible co-occurring mental health difficulties (as per endorsed mental health domain symptoms) has not been explored. Veterans living in NI have likely experienced a variety of lifetime traumas, many of which are specific to the NI landscape, thus it would be useful to explore which specific traumatic exposures were associated with specific comorbid mental health domains. Extending Spikol and colleagues’ [29] work, this exploratory study aimed to investigate both co-occurring trauma exposure and co-occurring mental health domain symptoms, and the relationship between them. It is also important to include alcohol as a mental health domain due to the military’s engrained relationship with it [20]. Co-occurring trauma exposure was defined as the endorsed range of distinctly different lifetime traumatic events as opposed to summative traumatic event exposure. Co-occurring mental health refers to a potential comorbidity of symptoms associated with six mental health domains: alcohol, anxiety, depression, PTSD, C- PTSD and dissociation. It was expected that defined co-occurring traumatic events would predict specific co-occurring mental health domain symptoms and that demographic factors (UDR service, worst trauma being military specific, age, gender) would also predict co-occurring mental health domain symptoms.

## Methods

### Sample data

Eligible data from *n* = 609 participants was extracted from the NIVHWS, a cross-sectional, self-report survey consisting of a range of sociodemographic, health/mental health, and well-being measures. The NIVHWS ran from December 2017 to June 2019. Total responses were *N* = 3,521 but *n* = 2,105 failed to provide consent, *n* = 78 gave consent but answered no questions, *n* = 8 gave implausible answers (e.g., joined at age five), and *n* = 1 was a test entry. Partial responses (80% complete) from *n* = 1,329 veterans were inspected; missingness patterns predominantly related to whole scales versus scale items. It was surmised that missingness was probably the result of the overall survey length (90 min). The remaining data (*n* = 609) was subjected to maximum-likelihood estimates, whereby expectation-maximisation (EM) algorithmic calculations were performed to estimate missing values in the process of obtaining model parameter estimates [30, 31].

The complete data set of *n* = 609 and the *n* = 1,329 partial responses were compared for demographic differences. Statistically, participants from the reduced data were more likely to endorse ‘other’ religion (69.0%/31.0%), more likely to have a diploma (61.2%/38.8%), bachelor’s degree (65.8%/34.2%), or doctorate (70%/30%), less likely to endorse full-time (40.2%/59.8%) or part-time employment (45.0%/55.0%) and were less likely to endorse Army service (32.5%/87.1%). Partial, meaningful responses indicated a NI veteran national response rate of approximately 2.22 − 3.32%.

Ethical approval was granted by Queen’s University Ethics Committee (EPS 19_156). All participants provided informed consent electronically.

### Measures

Sociodemographic data included gender, age, relationship status, ethnicity, religion, education, employment, and a range of military service-based variables (Table 1).

**Table 1:** Demographic information for *n* = 609

		N (%) *(M/S)*
*Age*		*55.26 (10.61)*
*Gender*	Male	546 (89.66)
	Female	63 (10.34)
*Relationship status*	Married or living with partner	448 (73.81)
	Separated or divorced	101 (16.64)
	Widowed	24 (3.95)
	Single or never married	34 (5.60)
*Ethnicity*	White	605 (99.67)
	Mixed/Other	2 (0.33)
*Religion*	No religion	134 (22.04)
	Protestant	371 (61.02)
	Catholic	47 (7.73)
	Other Christian	36 (5.92)
	Other	20 (3.29)
*Highest level of education*	Left school with no qualifications	96 (15.82)
	GCSE grade D-G/ NVQ level 1	50 (8.24)
	GCSE grade A-C/O-Levels	108 (17.79)
	A Levels	59 (9.72)
	Certificate of higher education	42 (6.92)
	Diploma of higher education	74 (12.19)
	Undergraduate degree or equivalent	73 (12.03)
	Master’s degree or post-graduate certificate/diploma	60 (9.88)
	Doctoral degree or Level 8 diploma	7 (1.15)
	Other	38 (6.26)
*Current employment status*	Unemployed	23 (3.78)
	Self-employed	41 (6.73)
	Employed full-time	279 (45.81)
	Employed part-time	48 (7.88)
	Student	11 (1.81)
	Unable to work	60 (9.85)
	Retired	134 (22.00)
	Medically retired	58 (9.52)
	Other	15 (2.46)
*Branch of service*	Royal Navy	40 (6.57)
	Royal Marines	7 (1.15)
	Army	527 (86.54)
	Royal Air Force	35 (5.75)
*Currently a Reservist*	Yes	56 (9.20)
	No	553 (90.80)
*Regular Forces service yrs*	Less than 1 year	5 (0.83)
	1–5 years	93 (15.35)
	6–10 years	144 (23.76)
	11–15 years	87 (14.36)
	16–20 years	64 (10.56)
	21–25 years	117 (19.31)
	26 + years	47 (7.72)
*Age at joined Armed Forces (M/SD)*		*18.47 (2.95)*
*Worst event due to miliary service*	Yes	325 (54.30)
	No	273 (45.70)
*Served in the UDR*	Yes	277 (45.48)
	No	332 (54.52)
*Mental health domain measures*	Audit alcohol scores	*7.34 (6.89)*
*DSS and ITQ do not provide M/SD	GAD-7 anxiety scores	*7.43 (6.89)*
	PHQ depression scores	*9.37 (8.29)*

*M/SD* = mean/standard deviation

Lifetime traumatic exposure was assessed by 16 items: 12 items were extracted from the Stressful Life Events Screening Questionnaire (SLESQ; [32]), and the remaining four were adapted from the Life Events Checklist for DSM-5 (LEC-5; [33]. Participants were instructed to indicate if they had experienced each trauma across their lifetime (yes = 1; no = 0), with the phrasing “other than experiences already covered…” to indicate the traumas were discreet incidents. Scale reliability for this population was α = 0.69. Supplementary file A describes the percentage of participants who endorsed experiencing each traumatic event.

Symptoms of hazardous alcohol use were assessed via the ten-item Alcohol Use Disorders Identification Test (AUDIT; [34]). Example item: “How often do you have six or more drinks on one occasion?” Eight items are scored 0 = never; 1 = less than monthly; 2 = monthly; 3 = weekly; 4 = daily/almost daily, and one item assesses the number of drinks consumed (0 = 1/2; 1 = 3/4; 2 = 5/6;3 = 7–9; 4=>=10). Item 10 is scored 0 = no; 2 = yes, not in the last year; 4 = yes, during the last year. Scores range 0–40, with higher summed scores indicating greater risky/hazardous drinking. The suggested scale cut-off for ‘probable risky drinker’ is = > 8 [34]. Reliability for this population was α = 0.87.

Symptoms of anxiety were assessed via the seven-item General Anxiety Disorder Assessment (GAD-7; [35]). Example item: “Over the last two weeks how often have you been bothered by trouble relaxing?” Scoring was 0 = not at all; 1 = several days; 2 = more than half the days; 3 = nearly every day. Scores ranged 0–21, with higher summed scores indicating worse anxiety. The suggested scale cut-off for probable anxiety is = > 10 [35]. Reliability for this population was α = 0.96.

Symptoms of depression were assessed via the nine-item Patient Health Questionnaire (PHQ; [36]). Example item: “Over the last two weeks how often have you been bothered by feeling down, depressed, or hopeless?” Scoring was 0 = not at all; 1 = several days; 2 = more than half the days; 3 = nearly every day. Scores ranged 0–21, with higher summed scores indicating worse depression. The suggested scale cut-off for probable depression is = > 10 [37]). Reliability for this population was α = 0.95.

Symptoms of PTSD and Complex PTSD (C-PTSD) were assessed via the International Trauma Questionnaire (ITQ; [38]). Example items: “How much have you been bothered by being super alert?”; “I feel numb or emotionally shut down”. Scoring was 0 = not at all; 1 = a little bit; 2 = moderately; 3 = quite a bit; 4 = extremely. Scores ranged from 0 to 36 across each of the PTSD and disturbance of self (DSO) dimensions. Caseness for PTSD and C-PTSD is algorithm-based, according to meeting the ‘moderate’ threshold across the PTSD/DSO scale dimensions. The moderate symptom threshold must be met for all eight dimensions spanning PTSD and DSO for C-PTSD, but only the four PTSD dimensions for PTSD [38]. A person is defined as meeting the symptomatic criteria for PTSD or C-PTSD, not both. Separate PTSD and C-PTSD caseness scores were used as individual predictor variables in the analysis. Reliability for the complete scale was α = 0.98.

Symptoms of dissociation were assessed via the Dissociative Symptoms Scale (DSS; [39]. Example item “Problems you may have experienced in the past week such as things around me seemed strange”. Scoring was 0 = not at all; 1 = once or twice; 2 = almost every day; 3 = about every day; 4 = more than once per day. Scores ranged from 0 to 80. To create a cut-off for probable caseness the scale authors suggested adding the sample mean to the 1.5 times inflated standard deviation; =>37.13. Reliability for the complete scale was α = 0.97.

All mental health domain assessment measures were self-report measures, and have been previously used in military, veteran or trauma-affected populations [16, 39–45]. Supplementary file B describes how many participants reached cut-off values for each mental health domain scale.

Military demographics included: a participant’s worst trauma being military-specific and UDR Service. ‘Worst trauma’ was assessed as “Was your worst traumatic experience related to your military experience?” (yes/no). UDR service was assessed as “Did you serve in the UDR?” (yes/no) and was used as an indicator of being exposed to elevated traumatic events both inside and outside employment. Military-specific trauma exposure and/or UDR service could account for the effect of general military service, or the experience of serving within one’s own neighbourhood, on poorer mental health. UDR service and worst trauma being military were binary variables (yes = 1; no = 0),

Demographic variables of age and gender were included as predictors of mental health domain class membership. Gender has produced a range of findings relative to mental health; also being younger has frequently indicated poorer mental health in veterans [22]. Age was a continuous variable and gender was binary, as no other genders were endorsed in the data of *n* = 609 (female = 1; males = 2).

### Data analysis

An automated three-step latent class analysis (LCA) was conducted to identify (1) groups of participants that shared latent properties concerning their responses to trauma experiences and, separately, mental health domain symptom scores, and (2) the subsequent relationship between trauma class membership and mental health domain symptom class membership, while factoring auxiliary variables [46, 47].

Initially, two LCAs were conducted: one based on the binary trauma exposure scores (1 = yes; 0 = no), and the second on dichotomized mental health domain symptom cut-off scores (1 = probable; 0 = not probable). The following goodness-of-fit indices were considered to determine an optimal class membership solution for each LCA: Akaike Information Criterion (AIC; [48]), Bayesian Information Criterion (BIC; [49]) and sample-size adjusted BIC (SSABIC; [50]). Lo-Mendel-Rubin Adjusted Likelihood Ratio Test (LMR-LTR; [51]) and bootstrapped LMR scores (BLMR; [52]) were also considered, together with how appropriate, interpretable and meaningful the classes were [46].

As the analysis considered mental health domain symptom class membership as a function of trauma exposure class membership (and other auxiliary variables), the three-step LCA included a logistic regression that used mental health domain symptom class membership as a categorical outcome. The largest mental health domain symptom class was used as the reference group. This analysis utilized the automated three-step approach in Mplus, as it was deemed appropriate for exploratory-only analysis [47, 53]. Initial data cleaning, EM calculations, scale scoring and reliability analysis were conducted in SPSSv25 [30, 31].

## Results

### Descriptive statistics

The sample cohort is described as 89.66% male, 99.67% white, having a mean age of 55.26 (*SD* = 10.61), with 72.73% 45 + years. Those who endorsed being married/living with a partner were 73.81%, 45.81% were employed full-time, and 61.02% identified as Protestant. Regarding military-specific demographics, 86.54% endorsed Army service and 9.20% were currently in the Reserves. Approximately half the cohort (45.48%) identified as having served with the UDR/Royal Irish, and 54.30% considered their worst traumatic experience was linked to military-specific service.

The five most commonly occurring single traumas were experiencing a fire/explosion (80.30%), being present when another was killed/harmed (61.74%), someone close died of unnatural causes (50.90%), exposure to gruesome death/harm (50.57%), and being threatened with a weapon (46.96%). Regarding probable mental health caseness, according to mental health domain symptom indicators, 40.72% scored as having probable depression, 36.78% had probable hazardous alcohol use, 32.50% had probable anxiety, 21.84% probable C-PTSD, 9.36% probable dissociation and 4.60% probable PTSD.

### Latent class analyses (LCA)

Models of two to six classes were run and fit statistics provided a range of model indices. Regarding the trauma endorsements best-class solution, the BIC value was at its lowest at the three-class solution which indicates a good model fit [49]. Although a weaker predictor, the AIC was also aligned at three classes. The LMR-LTR value indicated a three-class model was optimal due to it being the first instance of a statistically significant *p*-value [51]. The SSABIC is reported as being a strong indicator of optimal class solution where classes are uneven [52], which was relevant for the trauma exposures LCA. As the SSABIC value failed to align with any other meaningful indicators of good model fit the four-class solution suggested by the SSABIC value was disregarded. Also, despite BLMR values being a strong indicator of model fit [52, 54], these were disregarded as meaningful as all *p*-values were statistically significant. Regarding, the mental health domain symptom best-class solution, lowest AIC, BIC and SSABC values indicated a three-class solution was optimal. Although the LMR-LRT value suggested a four-class model, the first statistically significant BLMR value indicated a three-class model was optimal. Both three-class solution models seemed logical and conceptually meaningful (see Tables 2 and 3).

**Table 2:** Fit statistics for 2–6 class models of trauma responses

No of classes	Loglikelihood	Parameters	AIC	BIC	SSABIC	Entropy	LMR-LRT	BLMR (*p*)
2	-5156.134	43	10398.268	10587.976	10451.461	0.665	0.000	0.000
3	-5035.832	65	**10201.664**	**10488.432**	10282.072	0.764	**0.000**	0.000
4	-4999.352	87	10172.704	10556.532	**10280.326**	0.800	0.387	0.000
5	-4971.221	109	10160.442	10641.330	10295.279	0.753	0.249	0.000
6	-4940.467	131	10142.934	10720.882	10304.986	0.876	0.091	0.000

The bold values are the scores associated with optimal class solution as per the individual indicators

**Table 3:** Fit statistics for 2–6 class models of mental health domain symptom responses

No of classes	Loglikelihood	Parameters	AIC	BIC	SSABIC	Entropy	LMR-LRT	BLMR (*p*)	
2	-1429.627	13	2885.254	2942.608	2901.336	0.926	0.000	0.000	
3	-1385.454	20	**2810.907**	**2899.143**	**2835.648**	0.873	0.000	**0.000**	
4	-1379.057	27	2812.113	2931.232	2845.514	0.894	**0.015**	0.375	
5	-1373.483	34	2814.966	2964.968	2857.027	0.914	0.000	0.098	
6	-1371.601	41	2825.202	3006.087	2875.921	0.852	0.634	0.666	

The bold values are the scores associated with optimal class solution as per the individual indicators

#### A model of co-occurring, trauma-specific classes

Class 1 was the smallest with *n* = 66 (10.84%) participants. The highest level of probability was associated with ‘fire or explosion’ (0.87). Other combat/conflict-related traumas having a higher probability for Class 1 members were ‘close one died of unnatural causes’ (0.80), ‘exposure to gruesome details’ (0.79), ‘present when another was killed/harmed’ (.0.68), and ‘threatened with a weapon’ (non-mugging) (0.58). Sexual-interpersonal traumas endorsed as having higher probabilities were ‘ever physically assaulted’ (0.82), ‘touched inappropriately’ (0.84), ‘caregiver physically abusive’ (0.69), ‘physically forced sexual act’ (0.6) and ‘tried to force sexual act’ (0.46). As Class 1 members had a greater likelihood of experiencing higher levels of combat, conflict, and sexual-interpersonal traumatic events this class was labelled the ‘High Multi Trauma’ class.

Class 2 was the largest (*n* = 290, 47.62%). The highest probability of traumatic event endorsements was ‘experiencing fire/explosion’ (0.97), ‘present when another was killed/harmed’ (0.88), ‘exposure to gruesome details/harm’ (0.86), and ‘threatened with a weapon (non-mugging)’ (0.70). There were two moderately endorsed trauma items; ‘close one died from unnatural causes’ and ‘ever physically assaulted’. Conversely, ‘caused death/injury to another’ showed moderate endorsement (0.34), yet more in Class 2 endorsed causing death/injury to another than the other two classes. The pattern of endorsements describing Class 2 is predominantly aligned with combat and conflict-type situations, thus Class 2 was labelled the ‘High Combat Conflict’ class.

**Fig. 1:**
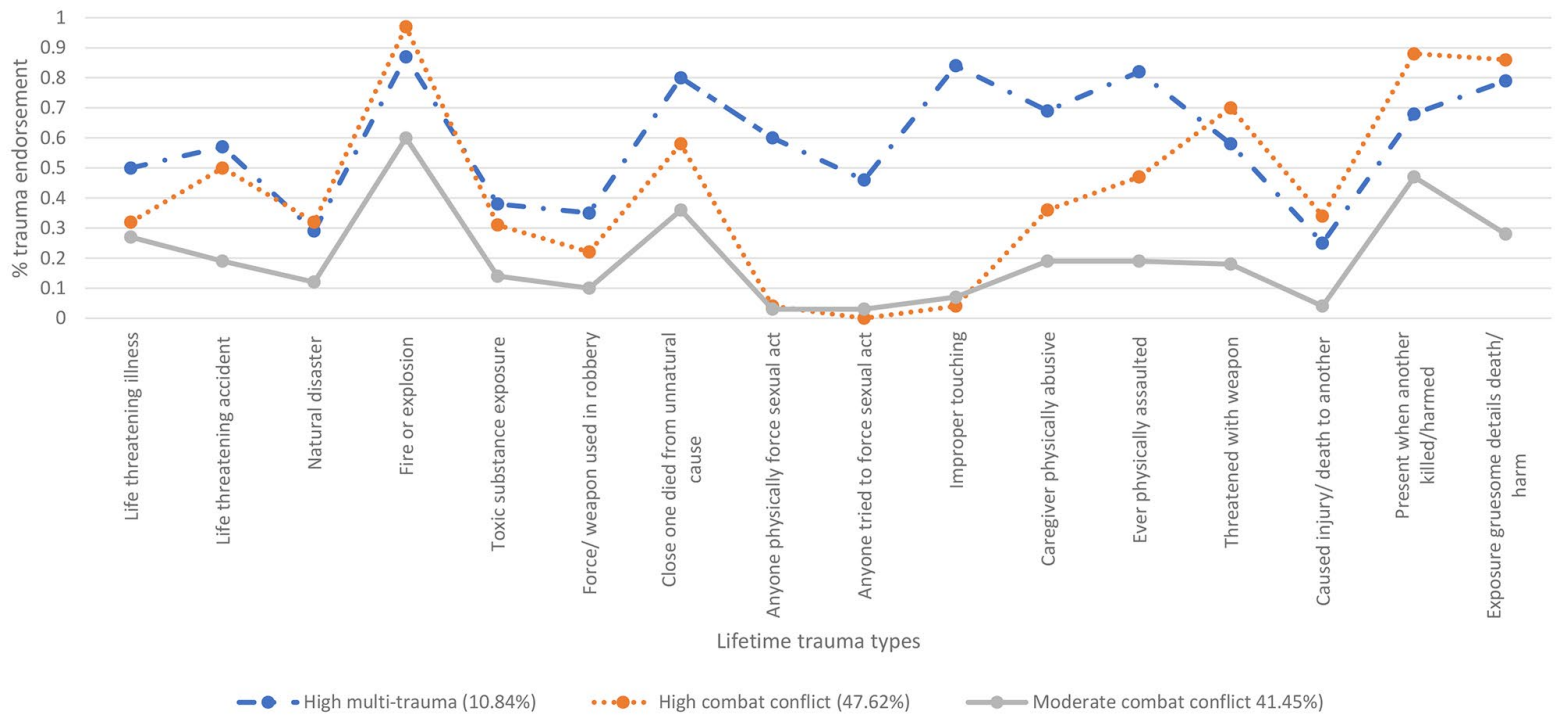
Three-class LCA solution based on endorsements of 16 lifetime traumas

Class 3 was the second largest (*n* = 253, 41.54%), with members showing the lowest probabilities of endorsing traumatic events compared to other classes. However, some combat/conflict traumatic events scored a moderate to above moderate probability of being experienced. ‘Experiencing a fire’ had the highest probability (0.60), followed by ‘present when another was killed/harmed’ (0.47), and ‘close one died of unnatural causes’ (0.36). All other traumatic events had a relatively low endorsement. Class 3 was labelled the ‘Moderate Combat Conflict’ class. See Fig. 1 for the three-class model of 16 traumatic events, and Supplementary Materials C for probability scores.

#### A model of co-occurring mental health and alcohol-specific classes

Class 1 (*n* = 118, 19.38%) endorsed reporting higher levels of symptoms linked to anxiety, depression and C-PTSD, together with moderate levels of dissociation and alcohol. The class was referred to as the ‘High Co-occurring Mental Health’ class.

Class 2 (*n* = 150, 24.63%) members endorsed reporting higher levels of symptoms linked to depression, with a moderate symptom level being linked to anxiety and alcohol. This class reported PTSD symptoms but at low levels. This class was referred to as the ‘High Depression Moderate Anxiety/Alcohol’ class.

**Fig. 2:**
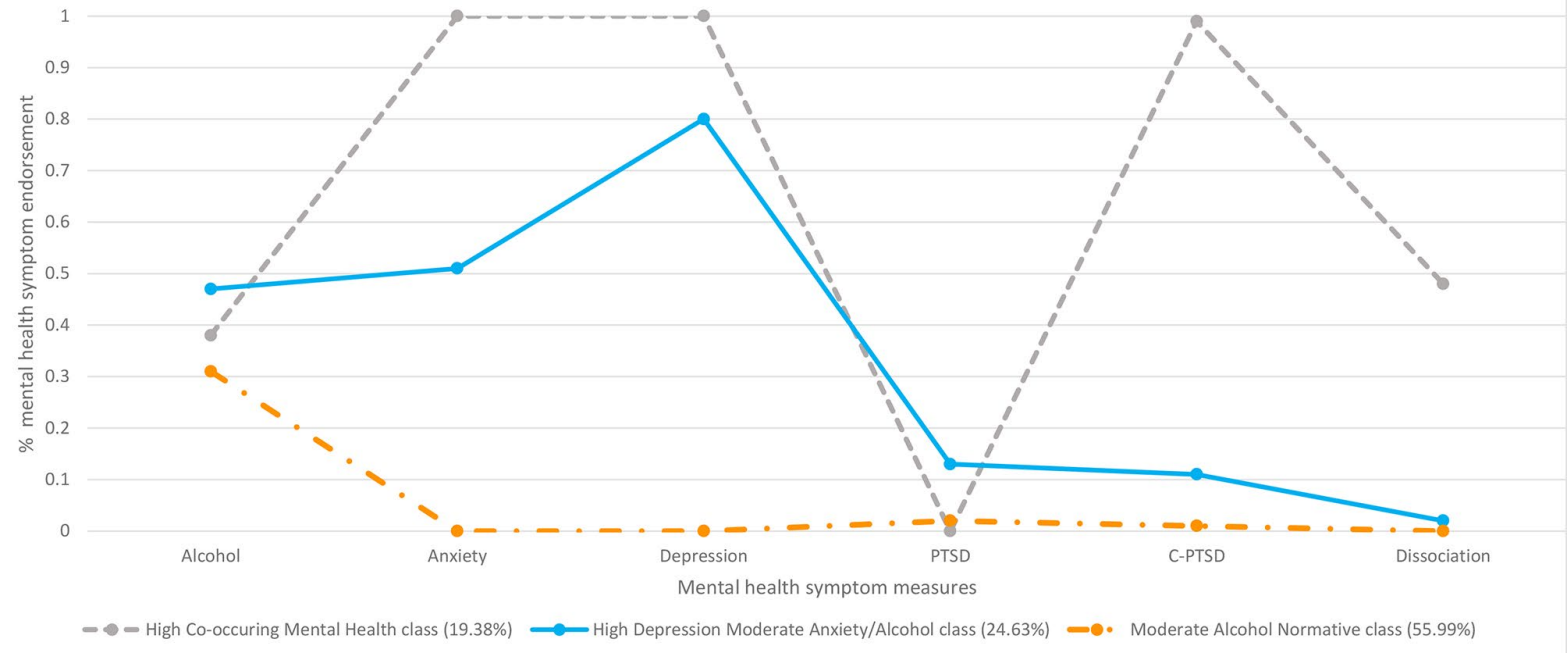
Three-class LCA solution based on endorsements of co-occurring mental health domain symptoms

Class 3 (*n* = 341, 55.99%) was the largest class and participants endorsed low/no likely mental health domain difficulties, with moderate alcohol issues. This class was referred to as the ‘Moderate Alcohol Normative’ class. See Fig. 2 for the three-class model of mental health symptoms, and Supplementary Materials D for probability scores.

### Regression

A multinomial logistic regression was modelled into the three-step LCA to explore mental health domain symptom class membership as a function of trauma exposure class, and other auxiliary variables (Table 4).

**Table 4:** Full multinomial regression of trauma exposure class predicting mental health domain symptoms class

	95% CI for Odds Ratios	
	High Co-occurring Mental Health class vs. Moderate Alcohol Normative class	High Depression Moderate Anxiety/Alcohol vs. Moderate Alcohol Normative class
High Combat Conflict Trauma class (yes)	0.641* (0.419 − 0.982)	1.101 (0.694–1.469)
High Multi Trauma class (yes)	2.506*** (1.458–4.308)	1.843* (1.073–3.165)
Moderate Combat Conflict Trauma class (yes)	0.261*** (0.162 − 0.419)	0.687* (0.473 − 0.999)
UDR service (yes)	2.992*** (1.880–4.760)	1.630* (1.092–2.435)
Worst trauma military specific (yes)	2.553*** (1.548–4.213)	1.168 (1.092–2.435
Age	0.986 (0.968–1.004)	0.976* (0.961 − 0.991)
Gender female (versus male)	1.227 (0.548–4.213)	1.690 (0.869–3.286)

Note *R*^2^ = 0.0851 (McFadden) 0.0545 (Cox & Snell), 0.113 (Nagelkerke), Model *X*^2^  = 102, *p* < .001. *p,0.05, **p < .01, ***p < .001

Mental health class membership was found to be a function of trauma class. High Multi Trauma class assignment (versus not being assigned to that class) significantly increased the likelihood of High Co-occurring Mental Health class assignment by 251% (*OR* 2.506, *CI* 1.458–4.308) or High Depression Moderate Anxiety/Alcohol class assignment by 184% (*OR* 1.843, *CI* 1.073–3.165) (compared to the Moderate Alcohol Normative class assignment).

An inverse relationship was noted regarding the two Combat Conflict classes and mental health domain class membership. High Co-occurring Mental Health class assignment significantly decreased by 35.9% (compared to Moderate Alcohol Normative mental health assignment) as a function of High Combat Conflict class membership (versus not being in that class) (*OR* 0.641, *CI* 0.491-0.982) and reduced by 73.9% due to Moderate Combat Conflict class membership (versus not being in that class) (*OR* 0.261, *CI* 0.162-0.419). Moderate Combat membership also reduced the likelihood of High Depression Moderate Anxiety/Alcohol class assignment by 31.3% (versus Moderate Alcohol Normative class) (*OR* 0.687, *CI* 0.473-0.999).

Regarding miliary-specific demographic predictors, the effect of being in the UDR (versus not) had a significant effect on mental health domain symptom class membership. Endorsing UDR service increased the likelihood of High Co-occurring Mental Health class assignment by 299% (*OR* 2.992, *CI* 1.880–4.760) and High Depression Moderate Anxiety/Alcohol class assignment by 163% (*OR* 1.630, *CI* 1.092–2435) (versus Moderate Alcohol Normative mental health class assignment). Endorsing one’s worst trauma as being military-specific (as opposed to another cause) increased the likelihood of High Co-occurring Mental Health class assignment by 255% (*OR* 2.553, *CI* 1.548–4.213) (versus Moderate Alcohol Normative class membership). UDR membership and military-specific trauma were the largest significant predictors of mental health domain symptom class membership.

Age was the final significant predictor of mental health domain symptom class assignment, yet the effect size was small. As age increased the likelihood of being assigned to the High Depression Moderate Anxiety/Alcohol class (relative to the Moderate Alcohol Normative mental health class) reduced by 2.4% (*OR* 0.976, *CI* 0.961-0.991).

## Discussion

The current study explored the landscape of co-occurring traumatic events and separately, co-occurring mental health domain symptoms. Subsequently, it was assessed whether specific co-occurring mental health domain symptoms were a function of defined co-occurring traumatic events and/or certain demographic factors. Using trauma exposure classes to predict co-occurring mental health symptoms was a novel approach across veteran-specific literature; these findings are to be interpreted as exploratory only.

Regarding co-occurring trauma exposure classes, this study found three classes that were described as High Multi-Trauma class, High Combat Conflict Trauma class and Moderate Combat Conflict class. Finding a smaller class with high exposure to all traumatic events was consistent with other veteran-specific studies [55, 56]. However, noting that approximately 90% of participants, across two trauma exposure classes, endorsed moderate to high levels of many combat and conflict-related traumatic events was unique to this study. Rossi and colleagues’ [57] study did report that approximately 25% of US veterans, some of whom lived in high-crime areas, were classified as ‘High Combat High Community Violence’ class members. Rossi et al., surmised that community violence was potentially linked to ‘occupation’ (i.e., emergency responders) or community hazards, but it was unclear what community violence consisted of. Whereas this current study defined specific traumatic events as occurring within an NI community context. Comparable research conducted by Gaska and Kimerling, and Davis et al. [55, 58], that included veteran responses to combat-related assessments found no combat trauma-specific class within the examined data. Although it was possible to determine during this current study that traumatic events occurred within the NI community, it was not defined whether traumatic experiences occurred during or outside work contexts.

When assessing the mental health domain symptoms, finding a three-class solution aligned with the existing body of evidence. Veteran or military-specific literature frequently reports finding a three or four-class solution. The three-class model found by Spikol and colleagues was maintained [29] after adding four additional mental health domains to PTSD and C-PTSD. Byrne et al., [59] and Contractor et al., [60] both reported three-class solutions within PTSD or PTSD and co-occurring substance use symptom indicators, in veteran and trauma-exposed populations. Contractor and colleagues noted a clear relationship between depression and two out of three PTSD/substance classes. Anxiety was also related to a high PTSD/high substance class, which aligns with this current study’s findings. Bryne and colleagues’ study found alcohol use disorder was linked to all three PTSD classes whereas depression was only significantly related to one PTSD (dysphoric symptoms) class, which differs from this current study.

The finding of higher rates of depression across two classes in this current study could be explained by the military or veteran disposition. Bartone and Homish [61] describe a veteran’s preference to be ‘hardy’ and ‘emotionally closed off’ as often contributing to depression. Likewise, it was (and still is) predominantly the case that NI veterans tend to be hardy, closed-off, and in a constant state of hypervigilance to manage their own threat perception. Additionally, evidence suggests that anxiety over re-occurring traumatic events mediates depression [62]. Fears over pending trauma in the NI context [25] could account for the high presence of anxiety and depression in both classes related to poorer mental health in this study.

Two concerning trends in this study are worth highlighting: first, one class representing nearly 20% of the sample endorsed experiencing mental health domain symptoms at moderate to high levels, across five measures. While this research is exploratory only, a finding such as this warrants further investigation, as it potentially indicates many in NI could be struggling with function impairment and poor quality of life. Murphy and colleagues [14] did suggest that poor mental health rates for NI veterans are likely, partially attributable to the poor mental health resources available. This may partly explain why a disproportionately large number of NI veterans have accessed Combat Stress compared to veterans from the other three UK nations [23]. Barriers to care in NI were found to predict worsening mental health for veterans living there [24], and a general reluctance to help-seeking exhibited by veterans no doubt compounds mental health difficulties [63, 64].

A reluctance to help-seek, and limited access to support, may go some way to account for the second concerning trend: symptoms associated with a moderate risk of alcohol use across appearing in all three mental health domain symptom classes. This is perhaps unsurprising given the military’s historic ‘reliant’ relationship with alcohol [20]. Yet, excessive alcohol use was also found in the class associated with general mental health wellness. This can be interpreted to mean that even if a veteran experienced improved mental health symptoms, and moved to the Normative mental health domain symptom class, they would still be at risk of experiencing hazardous alcohol use. If patterns of alcohol use are not related to poor mental health domain symptoms, they could be determined by factors such as cultural norms surrounding Irish and military identity [20, 65]. Thus, this is another finding that would benefit from further exploration.

Although classes of poorer mental health have been identified across extant literature, the majority of participants do endorse experiencing lower levels of mental health difficulties, which is mirrored in this study. The largest class (55.99%) had a low-to-no probability of experiencing symptoms associated with anxiety, depression, PTSD, C-PTSD or dissociation domains. These findings may be due to conceptualizing trauma as ‘not that traumatizing’ [66], or veterans experiencing a period of post-traumatic growth [13, 67]. Other factors, such as having good quality social support, appear to have had a positive effect on veteran mental health [22, 68].

When assessing whether mental health domain symptom class membership was a function of trauma exposure class, it was found that UDR service had a significant, unique impact on being assigned to a poorer mental health domain symptom class. The odds of being assigned to the High Co-occurring Mental health class increased by approximately 300%, compared to the Moderate Alcohol Normative class. Although UDR service had a predictive effect on being in the High Depression Moderate Anxiety/Alcohol class (versus Moderate Alcohol Normative class) it was lower. Endorsing one’s worst trauma as military-specific was also particularly predictive of High Co-occurring Mental health class membership (compared to the Moderate Alcohol Normative class). Taken together, the UDR who experienced their worst traumatic event in their own community have the highest risk of experiencing the worst mental health symptoms across a range of domains. This is likely due to attempting to manage past traumatic experiences with limited resources and barriers to care [14, 24], while symultaneously living with a constant concern over pending or future trauma [25]. The former UDR may be the least likely to seek help, which could exacerbate a range of mental health symptoms [18].

High Co-occurring Mental health and High Depression Moderate Anxiety/Alcohol class assignment, compared to Moderate Alcohol/Normative class assignment, were predicted by being in the High Multi-Trauma class (versus not being in it). This was perhaps expected as the High Multi-Trauma class included interpersonal trauma of a sexual nature, which has been found to be particularly predictive of poorer mental health in veterans [69]. However, the finding that both combat/conflict classes (compared to not being in that class) had a significant predictive relationship with the Moderate Alcohol Normative class versus the poorer mental health domain symptom classes was unique to this study. It was expected that the High Combat/Conflict class would have a greater predictive effect on being assigned to a poorer mental health domain symptom class (compared to the Moderate Alcohol Normative class) as previous studies have found a relationship between high levels of combat trauma and poorer mental health outcomes [55, 57].

A possible explanation could be that many participants with a greater likelihood of being assigned to a conflict class, including the High Conflict/Combat class (compared to not being in that class) were also assigned to the lower mental health difficulty class (compared to the poorer classes). Given that the mental health latent class analysis in this study found concerning levels of symptoms, and approximately 48% were categorised as experiencing high levels of traumatic exposure experiences, it is suggested that further exploration is needed. As well as returning to the quantitative data, perhaps a qualitative approach could be considered with veterans living in NI. A qualitative approach could gain greater insights into participants’ thoughts and feelings towards conflict/combat situations. Participants could be asked what specifically, perhaps collectively, contributed to their mental health outcomes.

### Limitations and strengths

It is not possible to say the NIVHWS data represents NI veterans due to the sample size used within this study. Females and officers were underrepresented, which was to be expected as the military is generally non-officer, male-dominant. Also, general criticisms surrounding cross-sectionality and the use of secondary, self-reported data apply. It is acknowledged for the analysis there was a chance of compounding error associated with including two LCAs in the same model, which was partially mitigated with an automated three-step LCA. Automation may lack some robustness when accounting for the full effect of any additional variables on class membership. While the automated approach is considered acceptable for exploratory research, if this study was extended it would be advisable to conduct a fully manualized three-step LCA [47]. It was considered that this method was suitable for this study as it was exploratory only, yet if this research was extended to make clinical inferences more robust models of measurement and analysis are recommended.

Despite the limitations, it should be recognized that this study was based on data collected via the first national veteran survey to be administered within NI. To date, it is the most accurate NI veteran data available, collected in a non-clinical setting. Although this study focused on eliciting clusters of mental health-related symptoms, the information produced would be useful for medical professionals within a clinical diagnosis context or for clinicians engaging in further research. This study found some utility in the novel analytic method adopted, using latent class membership to predict the membership of another set of latent classes. The manualized 3-step model could be applied to other populations and in other contexts, to explore relationships between a range of co-occurring issues.

### Implications

It can be assumed that those who experience particularly poor co-occurring mental health domain symptoms will likely have the most complex needs and greater functional impairment. Trauma exposure-informed care should be delivered in a manner that considers the context in which the traumatic exposure occurred, which is most salient for veterans such as the former UDR. A veteran’s responses to enquiries of trauma exposure may pre-empt what co-occurring mental health difficulties they are experiencing, or the degree to which their mental health status is causing impairment. The opposite may also be true; mental health domain symptom clusters may be an indicator of traumatic events experienced, which is useful information for those who support people that struggle to discuss their trauma exposure. The wider implications infer that others experiencing high levels of multi-trauma, or events perceived as particularly traumatic, may be experiencing specific co-occurring mental health difficulties as indicated by their symptoms. Such difficulties are likely linked to trauma exposure that occurred within specific personal and cultural landscapes. Cultural landscapes need to be included within a trauma exposure-informed care program, to better understand and meet people’s needs.

## Conclusions

Defined co-occurring traumatic events did predict specific mental health domain symptom classes within a population of veterans resident in NI. High levels of multi-trauma were particularly predictive of experiencing high rates of symptoms linked to between three and five mental health domains. The UDR in particular could not escape traumatic event exposure in NI, which could account for the link between one’s worst trauma being military-specific, UDR service, and experiencing higher rates of co-occurring mental health domain symptoms. The UDR were also more likely to experience anxiety/depression symptoms, perhaps due to living with a constant threat of pending trauma exposure together with a greater reluctance to seek help. Hazardous alcohol use was present regardless of whether other poor mental health domain symptoms were experienced, which could be explained by both military and Northern Irish cultural factors.

While these data are not representative of the wider community of NI veterans, there is an indication that the veteran community could be experiencing poorer mental health compared to the other nations of the UK. Clinically, veterans in NI would benefit from support which factors its specific landscape and understands the cultural norms of hypervigilance and a need for privacy. Further research is warranted to clarify the mental well-being of veterans living in NI. Additionally, it would be useful to examine their actual help-seeking behaviours in the context of NI, and qualitatively explore their real-life experiences.

## Electronic supplementary material

Below is the link to the electronic supplementary material.

Supplementary Material 1

Supplementary Material 2

Supplementary Material 3

Supplementary Material 4

## Data Availability

The datasets used and/or analysed during the current study are available from the corresponding author upon reasonable request. It has not been deposited for public access as it is restricted data.
