# Supplementary material for: The relationship between co-occurring traumatic experiences and co-occurring mental health domains for veterans resident in Northern Ireland
Source: BMC Psychol. 2024 Oct 1;12:523. doi: 10.1186/s40359-024-01991-4 (PMC11446063; doi:10.1186/s40359-024-01991-4)
Supplement: Supplementary file 1 — Supplementary Material 1 [file 40359_2024_1991_MOESM1_ESM.docx]

**Supplementary Material A**

*The percentage (%) that endorsed a particular trauma*
