# Supplementary material for: The relationship between co-occurring traumatic experiences and co-occurring mental health domains for veterans resident in Northern Ireland
Source: BMC Psychol. 2024 Oct 1;12:523. doi: 10.1186/s40359-024-01991-4 (PMC11446063; doi:10.1186/s40359-024-01991-4)
Supplement: Supplementary file 2 — Supplementary Material 2 [file 40359_2024_1991_MOESM2_ESM.docx]

**Supplementary Material C**

*Probability scores associated with class membership as per trauma indicators*

|  | High multi-trauma (*n*=66/10.84%) | High combat conflict (*n*=290/47.62%) | Moderate combat conflict (*n*=253/41.45%) |
| --- | --- | --- | --- |
| Life threatening illness | 0.5 | 0.32 | 0.27 |
| Life threatening accident | 0.57 | 0.5 | 0.19 |
| Natural disaster | 0.29 | 0.32 | 0.12 |
| Fire or explosion | 0.87 | 0.97 | 0.6 |
| Toxic substance exposure | 0.38 | 0.31 | 0.14 |
| Force/ weapon used in robbery | 0.35 | 0.22 | 0.1 |
| Close one died from unnatural cause | 0.8 | 0.58 | 0.36 |
| Anyone physically force sexual act | 0.6 | 0.04 | 0.03 |
| Anyone tried to force sexual act | 0.46 | 0 | 0.03 |
| Improper touching | 0.84 | 0.04 | 0.07 |
| Caregiver physically abusive | 0.69 | 0.36 | 0.19 |
| Ever physically assaulted | 0.82 | 0.47 | 0.19 |
| Threatened with weapon | 0.58 | 0.7 | 0.18 |
| Caused injury/ death to another | 0.25 | 0.34 | 0.04 |
| Present when another killed/harmed | 0.68 | 0.88 | 0.47 |
| Exposure gruesome details death/ harm | 0.79 | 0.86 | 0.28 |
