# Supplementary material for: The relationship between co-occurring traumatic experiences and co-occurring mental health domains for veterans resident in Northern Ireland
Source: BMC Psychol. 2024 Oct 1;12:523. doi: 10.1186/s40359-024-01991-4 (PMC11446063; doi:10.1186/s40359-024-01991-4)
Supplement: Supplementary file 3 — Supplementary Material 3 [file 40359_2024_1991_MOESM3_ESM.docx]

**Supplementary file B**

*Number of participants that reached clinical cut-offs, as scored likely (1) or unlikely (0) for each mental health domain indicator*

| **Scales** | ***n (%) L*ikely experience (1)** | ***n* (%) Unlikely experience (0)** |
| --- | --- | --- |
| Alcohol - AUDIT | 224 (36.78) | 385 (63.22) |
| Anxiety - GAD-7 | 198 (32.50) | 411 (67.50) |
| Depression - PHQ | 248 (40.72) | 361 (59.28) |
| PTSD - ITQ | 28 (4.60) | 581 (95.40) |
| C-PTSD - ITQ | 133 (21.84) | 476 (78.16) |
| Dissociation - DSS | 57 (9.36) | 552 (90.64) |
