# Supplementary material for: The relationship between co-occurring traumatic experiences and co-occurring mental health domains for veterans resident in Northern Ireland
Source: BMC Psychol. 2024 Oct 1;12:523. doi: 10.1186/s40359-024-01991-4 (PMC11446063; doi:10.1186/s40359-024-01991-4)
Supplement: Supplementary file 4 — Supplementary Material 4 [file 40359_2024_1991_MOESM4_ESM.docx]

**Supplementary Material D**

*Probability scores associated with class membership as per mental health domain and symptom indicators*

|  | High Co-occurring Mental Health class (*n*=118/19.38%) | High Depression Moderate Anxiety/Alcohol class (*n*=150//24.63%) | Moderate Alcohol Normative class (*n*=341/55.99%) |
| --- | --- | --- | --- |
| Alcohol | 0.38 | 0.47 | 0.31 |
| Anxiety | 1.00 | 0.51 | 0.00 |
| Depression | 1.00 | 0.80 | 0.00 |
| PTSD | 1.00 | 0.13 | 0.02 |
| C-PTSD | 0.99 | 0.11 | 0.01 |
| Dissociation | 0.48 | 0.02 | 0.00 |
